# Supplementary material for: Adaptations in wing morphology rather than wingbeat kinematics enable flight in small hoverfly species
Source: eLife. 2025 Sep 30;13:RP97839. doi: 10.7554/eLife.97839 (PMC12483507; doi:10.7554/eLife.97839)
Supplement: Supplementary file 1. — (a) Results of analyses of variance (ANOVAs) testing the effect of sex and species on wing morphology parameters. (b) Phylogenetic signal computed on morphological and flight traits. Species number in the morphology and flight dataset is 28 and 8, respectively. (c) Results from multiple regressions testing correlations between the wingbeat kinematics parameters and body kinematics, expressed by flight speed and climb angle. (d) Results of phylogenetic generalised least squares (PGLS) regressions of additional wingbeat kinematic parameters against body mass. [file elife-97839-supp1.docx]

**Supplementary Tables**

**Supplementary File 1a.** Results of ANOVAs testing the effect of sex and species on wing morphology parameters.

| Variable | Factor | F-value | P-value |
| --- | --- | --- | --- |
|  |  |  |  |
| Body mass *m* |  |  |  |
|  | sex | 4.061 | **0.047** |
|  | species | 53.897 | **<0.001** |
| Second-moment-of-area *S*_2_ |  |  |  |
|  | sex | 11.592 | **0.001** |
|  | species | 38.489 | **<0.001** |
| Wingspan *R* |  |  |  |
|  | sex | 0.334 | 0.564 |
|  | species | 67.411 | **<0.001** |
| Wing chord *c̄* |  |  |  |
|  | sex | 6.202 | **0.014** |
|  | species | 71.933 | **<0.001** |
| Normalized second- moment of-area *S*_2_* |  |  |  |
|  | sex | 2.719 | 0.103 |
|  | species | 21.231 | **<0.001** |
|  |  |  |  |
| Bold indicates statistical significance |  |  |  |

**Supplementary File 1b.** Phylogenetic signal computed on morphological and flight traits. Species number in the morphology and flight dataset is 28 and 8, respectively.

| **Morphological traits** | | |  | **Flight traits** | | |
| --- | --- | --- | --- | --- | --- | --- |
|  | Blomberg’s K | P |  |  | Blomberg’s K | P |
| Body mass *m* | 0.81 | **0.004** |  | Wingbeat frequency *f* | 0.66 | 0.696 |
| Wingspan *R* | 0.66 | **0.005** |  | Stroke amplitude 𝐴_𝜙_ | 0.91 | 0.187 |
| Wing chord *c̄* | 0.72 | **0.004** |  | $\bar{\omega}$   \| Wing angular speed \| \| --- \| | 0.89 | 0.235 |
| Second moment of area *S_2_* | 0.59 | 0.071 |  | $\bar{\alpha}$   \| Angle-of-attack \| \| --- \| | 1.01 | 0.145 |
| Normalized second moment of area *S_2_** | 0.88 | **0.004** |  |  |  |  |
|  |  |  |  |  |  |  |
| Bold indicates statistical significance. | |  |  |  |  |  |

**Supplementary File 1c.** Results from multiple regressions testing correlations between the wingbeat kinematics parameters and body kinematics, expressed by flight speed and climb angle.

|  | Flight speed | | Climb angle | |
| --- | --- | --- | --- | --- |
|  | t value | P | t value | P |
| Wingbeat frequency *f* | 1.976 | 0.057 | -0.401 | 0.691 |
| Stroke amplitude 𝐴_𝜙_ | -0.037 | 0.971 | 0.602 | 0.552 |
| $\bar{\omega}$   \| Wing angular speed \| \| --- \| | 2.331 | **0.026** | 0.499 | 0.621 |
| $\bar{\alpha}$   \| Angle-of-attack \| \| --- \| | 0.721 | 0.477 | -0.451 | 0.655 |
| Bold indicates statistical significance. | |  |  |  |

**Supplementary File 1d.** Results of phylogenetic generalized least square (PGLS) regressions of additional wingbeat kinematic parameters against body mass.

|  |  | n | P | R^2^ | Intercept | Scaling factor  estimate [95% C.I.] |
| --- | --- | --- | --- | --- | --- | --- |
|  |  |  |  |  |  |  |
|  | Rotation amplitude *A*_θ_ | 8 | 0.301 | 0.25 | 4.692 | 0.016 [-0.011 – 0.044] |
|  | Deviation amplitude *A*_η_ | 8 | 0.942 | 0.07 | 0.492 | 0.022 [-0.551 – 0.595] |
|  | Peak stroke rate ϕ ̇_peak_ | 8 | 0.201 | 0.11 | 11.178 | -0.103 [-0.244 – 0.037] |
|  | Peak rotation rate θ ̇_peak_ | 8 | 0.901 | 0.03 | 11.488 | 0.014 [-0.203 – 0.232] |
|  | Peak deviation rate η ̇_peak_ | 8 | 0.247 | 0.14 | 10.308 | -0.216 [-0.546 – 0.114] |
|  |  |  |  |  |  |  |
